# Chloroform Versus Ether

**Published:** 1880-07

**Authors:** 


### Editorial.

---

### CHLOROFORM VERSUS ETHER.

The sad case of death under the influence of chloroform noted in our columns this month is but one of five which have been reported in the journals as occurring during the month of May from chloroform narcosis, and as we write, the telegraph brings the account of another death in this vicinity. In all of these cases it is claimed that the anæsthetic was administered by capable hands and with due caution. Not a few physicians, at least in this city, have a preference for chloroform, and we are accustomed to say that properly administered, there is no more danger in its use than with ether.

With our present knowledge are we justified in this conclusion? The advocates of chloroform certainly can not base their preference for its use upon authorities or statistics, for the long and painful record of valuable lives lost from the time of its introduction as an anæsthetic to the present day, has caused almost every student of the subject to take positive grounds against its general use.

The common opinion that the cause of the fatality from chloroform depends upon any particular mode of administration is not supported by a study of fatal cases, nor by a long series of experiments upon animals.

The case reported here is but one of many which show conclusively that chloroform will kill, when prior to administration the patient shows no trace of disease or other sign, by which the danger of death can be foretold. As to the difference of anæsthesia by ether and chloroform, Schiff arrived at the following conclusions after more than five thousand experiments:

"Ether paralyzes first the respiration and after that the blood vessels and the heart, while chloroform can paralyze the heart and blood vessels at once, without previously paralyzing the respiration; artificial respiration with the latter agent is then useless, as oxygenation has ceased. Compression of the abdominal vessels and lowering of the head may be of advantage. Chloroform can cause death at the first inspiration."

The irresistible argument, however, as to the merits of the two anæsthetics, is the result of later studies of statistics. From these we learn that chloroform destroys one person out of every twenty-five hundred who inhale it, while the deaths from ether are only one in twenty-three thousand two hundred and four. An analysis of the cases of death from ether upon which these statistics are based, show that more than one-half of them are fairly attributable to other causes than the anæsthetic, and when we consider how carelessly it is often administered, these results almost justify us in considering it an absolutely safe anæsthetic. With chloroform, on the contrary, we are fully satisfied that no amount of care or precaution, or mode of administration, or amount inhaled, will prevent in certain cases the fatal result. In the light of this experience, and in the face of these facts, is any physician justified in resorting to the use of chloroform in ordinary cases? That there are exceptional cases in which it is the better agent, we admit; but we hold that the physician assumes a solemn responsibility who selects the more dangerous anæsthetic only because more convenient in some respects to give, and pleasanter for the patient to take, and thus unnecessarily subjects his patient to a risk, however small, of losing his life.

